# Systematic review and meta-analysis of effectiveness of robotic therapy in the recovery of motor functions after stroke

**DOI:** 10.3389/fnhum.2025.1622661

**Published:** 2025-07-21

**Authors:** Mariyam Amirbekova, Tokzhan Kispayeva, Ausra Adomaviciene, Laura Eszhanova, Inna Bolshakova, Zhanna Ospanova

**Affiliations:** ^1^Institute of Life Sciences, Karaganda Medical University, Karaganda, Kazakhstan; ^2^School of Nursing Education, Karaganda Medical University, Karaganda, Kazakhstan; ^3^Rehabilitation Center “Neuron”, Karaganda, Kazakhstan; ^4^Department of Rehabilitation, Institute of Medical Sciences, Physical and Sports Medicine, Vilnius University, Vilnius, Lithuania; ^5^Department of Neurology, Medical University of Astana, Astana, Kazakhstan; ^6^Department of History of Kazakhstan and Socio-Political Disciplines, Karaganda Medical University, Karaganda, Kazakhstan

**Keywords:** robotic therapy, stroke recovery, meta-analysis, neurorehabilitation, motor functions

## Abstract

**Background:**

Stroke is a leading cause of adult disability worldwide, often resulting in persistent motor impairments. While conventional rehabilitation approaches often yield modest results, robotic-assisted therapy has emerged as a promising solution to enhance motor recovery. However, the impact of stroke phase (acute, subacute, chronic) and other clinical modifiers on the effectiveness of robotic rehabilitation remains underexplored.

**Methods:**

The protocol for this systematic review and meta-analysis was registered in PROSPERO under the registration number CRD420251038754. A systematic review and meta-analysis were conducted in accordance with PRISMA guidelines. The literature search was conducted using MEDLINE, PubMed, Cochrane Library, Scopus, Web of Science, and EMBASE. Risk of bias was assessed using the RoB 2.0. Primary outcomes included motor recovery, gait speed, and balance. A random-effects model (DerSimonian-Laird) was applied to calculate pooled standardized mean differences (SMD), and subgroup analyses and meta-regression were used to assess the influence of stroke phase, age, therapy duration, and combined interventions (e.g., virtual reality, mirror therapy).

**Results:**

Thirteen randomized controlled trials (RCTs) published between 2015 and 2025 were included, with a total of 424 post-stroke patients. Robotic therapy showed a moderate but statistically significant effect over conventional rehabilitation (SMD = 0.59, 95% CI: [0.33; 0.84], *p* < 0.001), with low-to-moderate heterogeneity (I^2^ = 30.5%). Subgroup analysis revealed the strongest effects during the subacute phase (SMD = 0.74) and acute phase (SMD = 0.75), while the chronic phase yielded limited improvement (SMD = 0.23). Younger age and a intervention duration of more than 6 weeks were associated with enhanced outcomes. Meta-regression indicated a trend toward reduced effectiveness with prolonged intervention duration (*β* = −0.134), although not statistically significant (*p* = 0.102). No publication bias was detected (Egger’s *p* = 0.56).

**Conclusion:**

Robotic-assisted therapy provides clinically meaningful improvements in post-stroke motor recovery. The findings support early stratification and personalization of rehabilitation programs based on stroke timing, age, and intervention intensity. Integration of robotic systems with virtual and cognitive components may further enhance neuroplasticity, leading to improved functional outcomes.

**Systematic Review Registration:**

https://www.crd.york.ac.uk/PROSPERO/view/CRD420251038754.

## 1 Introduction

Stroke is a leading cause of disability worldwide, as shown by global statistics highlighting its high prevalence and significant impact on patients’ quality of life. Each year, over 12 million new cases of stroke are reported worldwide, resulting in substantial economic and social burdens (Rakhimova et al., 2021). Moreover, stroke is often accompanied by persistent motor impairments, creating a critical need for effective rehabilitation strategies to restore lost motor functions. Modern medicine recognizes stroke not only as a clinical issue but also as a major challenge for healthcare systems, demanding the adoption of innovative treatment approaches (Kwakkel et al., 2023).

Traditional rehabilitation methods such as physiotherapy, kinesiotherapy, and occupational therapy remain the cornerstone of post-stroke recovery; however, their effectiveness is often limited. These approaches require a significant time and active patient participation—conditions not always feasible in routine clinical settings (Lee et al., 2022). Furthermore, conventional strategies frequently result in slow and incomplete recovery of motor functions, particularly among patients with severe impairments. Recent research indicates that standard rehabilitation techniques can only partially compensate for lost functions, leaving a considerable proportion of patients with long-term disabilities (Villafañe et al., 2018).

Given the limited efficacy of traditional approaches, increasing attention is being directed toward innovative technologies capable of enhancing the neurorehabilitation process. One such approach is robotic-assisted therapy, which has gained widespread clinical adoption in recent decades (O'Flaherty and Ali, 2024). Robotic systems enable the delivery of high-intensity, repetitive exercises with objective measurement of progress—an advantage over conventional rehabilitation methods. The use of robotic devices can help accelerate the neuroplastic processes that are necessary to restore motor function after a stroke (Singh et al., 2021).

Among the robotic systems used in rehabilitation, devices such as the Lokomat have received regulatory approval and are employed in numerous clinical centers. These systems ensure precise execution of movements, objective assessment of recovery dynamics, and the ability to tailor therapy protocols to the individual characteristics of each patient (Dehem et al., 2019). A number of studies have demonstrated that robotic-assisted interventions result in significantly better motor outcomes compared to conventional physiotherapy. For instance, a study by Zhang et al. (2024a, 2024b) showed that robotic therapy led to improved FMA-UE scores, indicating enhanced motor control recovery.

However, despite the promising outcomes, several critical questions remain unresolved and warrant further investigation. Importantly, many studies primarily focus on the recovery of upper limb function, while evidence regarding the impact of robotic therapy on lower limbs and gait is limited (Takebayashi et al., 2022). In addition, combined approaches that integrate robotic therapy with VR technologies have not been sufficiently studied. These hybrid interventions may produce a synergistic effects greater than either modality alone; however, their optimal parameters and long-term effectiveness remain a matter of ongoing debate (Inoue et al., 2022).

In robotic therapy, particular attention should be paid to the timing of rehabilitation initiation. Several studies suggest that brain neuroplasticity is most pronounced during the subacute phase of stroke, contributing to better recovery outcomes during this period (Kayabinar et al., 2021). At the same time, show high variability in response to therapy in their response to therapy, which may be associated with the high neurological instability characteristic of the early post-stroke stages. In patients with chronic stroke, despite ongoing reparative processes, the effects of robotic therapy are typically less pronounced, likely due to reduced neuroplastic potential (Louie et al., 2020).

Thus, the influence of stroke stage on the effectiveness of robotic therapy remains one of the central questions in neurorehabilitation. Stratifying data by subgroups—acute, subacute, and chronic stroke—enables a more nuanced evaluation of the time window in which robotic intervention is most effective (Ranzani et al., 2020). Such an approach not only refines clinical guidelines but also contributes to the development of personalized therapy protocols tailored to different patient populations. Previous research highlights substantial differences in therapeutic effects across stroke phases, highlight the importance of subgroup analysis (Torrisi et al., 2021).

With rapid advancements in rehabilitation technology, integrating evidence on robotic therapy with stroke recovery timelines is becoming increasingly important. This integration can not only improve rehabilitation outcomes but also reduce the risk of long-term disability in stroke survivors (Kolářová et al., 2022). The implementation of robotic therapy during the subacute phase may significantly accelerate the restoration of motor function, as supported by clinical trial data. Further research is needed to optimize outcomes by exploring how intensity and duration of interventions affect recovery (Veerbeek et al., 2017).

Recent studies have demonstrated that combining robotic devices with adjunct technologies improves outcomes. For example, the integration of VR not only stimulates motor processes but also enhances cognitive function, an essential component of comprehensive rehabilitation (Talaty and Esquenazi, 2023). These multifactorial approaches represent a promising direction; however, they require standardization and further validation through randomized controlled trials (Ranzani et al., 2020).

Given these challenges and opportunities, a systematic review is warranted—one that synthesizes existing research findings and identifies key modifiers influencing the effectiveness of robotic therapy.

*The aim of our study* is to comprehensively evaluate the effectiveness of robotic-assisted therapy in the neurorehabilitation of post-stroke patients and to determine how the timing of therapy initiation and patient age influence therapeutic outcomes.

Our work seeks to address existing gaps in the literature regarding the impact of stroke stage on the effectiveness of robotic therapy and to identify optimal intervention parameters for different patient populations.

The integration of advanced robotic technologies into clinical practice represents a promising direction in neurorehabilitation, with the potential to substantially enhance the restoration of motor function following stroke (Stinear et al., 2020). This approach supports the individualization of therapy in accordance with the current trends of personalized medicine (O'Flaherty and Ali, 2024).

The implementation of robotic-assisted therapy in clinical practice—particularly when applied within optimal time frames—opens new avenues in addressing the long-term consequences of stroke (Liu et al., 2025).

The novelty of this study lies in the comprehensive synthesis of randomized controlled trials evaluating the efficacy of robotic therapy in post-stroke rehabilitation (Picelli et al., 2024; Saarakivi et al., 2024; Zhou et al., 2022; Youn et al., 2024).

*Study objectives:*

1 To analyze RCTs on the impact of robotic-assisted therapy on motor function recovery.
2 To compare its effectiveness with conventional rehabilitation.
3 To evaluate the influence of therapy duration, stroke phase, and combined interventions.

## 2 Methods

The protocol for this systematic review and meta-analysis was registered in the International Prospective Register of Systematic Reviews (PROSPERO) under the registration number CRD420251038754.

### 2.1 Data extraction

This study involved a systematic review and meta-analysis of RCTs assessing the effectiveness of robotic-assisted therapy for restoring motor function in post-stroke patients. A comprehensive search was conducted across MEDLINE, PubMed, the Cochrane Library, Scopus, Web of Science, EMBASE. Search strategies combined keywords and Boolean operators such as “stroke rehabilitation” AND (“robotic therapy” OR “robot-assisted rehabilitation” OR “exoskeleton” OR “upper limb robot” OR “gait training robot”). Medical Subject Headings (MeSH) terms were used to enhance search sensitivity. Filters included: English language, randomized controlled trials, open-access availability, and a publication period from 2015 to 2025.

### 2.2 Quality assessment

All retrieved articles were imported into Zotero, where duplicates were automatically removed (Morgan, 2024). Two independent reviewers (MA and TK) screened titles and abstracts for relevance, followed by full-text review. Disagreements were resolved by discussion or, if needed, by a third reviewer (LE). The study followed PRISMA guidelines, ensuring methodological transparency. The screening process was illustrated using a PRISMA flow diagram (Page et al., 2021).

### 2.3 Inclusion and exclusion criteria

The inclusion criteria were as follows: (a) study type: we included randomized control trials in English, published between 2015 and 2025 (b) study population: all patients aged ≥18 years with a confirmed diagnosis of stroke (ischemic or hemorrhagic) and motor impairments (c) intervention: Studies in which the experimental group received robotic-assisted therapy as the primary rehabilitation method, and which included a control group receiving standard care or conventional therapy (d) full-text availability of the article (e) use of validated motor function assessment tools (e.g., FMA-UE, MAS, BBS, FAC, 10MWT). Exclusion criteria: (a) RCT protocols, systematic review of RCTs, secondary analysis of RCT data. (b) Studies with a small sample size (fewer than 10 participants in either group). (c) Articles lacking sufficient methodological or statistical information. (d) Studies conducted on participants under the age of 18. (e) Studies not published in English.

### 2.4 Data collection and quality assessment

Data were extracted using a standardized, pre-tested form. Extracted variables included participant age, stroke phase (acute, subacute, or chronic), intervention duration and frequency, type of robotic device (upper or lower limb), and use of adjunct therapies (e.g., VR or MT). Outcomes were measured using validated instruments: FMA-UE, MAS, BBS, FAC, and 10MWT.

The methodological quality of included studies was assessed using the Cochrane Risk of Bias 2.0 tool (RoB 2.0), which evaluates study design domains such as sequence generation, allocation concealment, blinding, data completeness, and potential bias sources (Crocker et al., 2023).

### 2.5 Statistical analysis

Statistical analyses were performed using RevMan 5.4 and Stata 16.0. For continuous outcomes, the mean difference (MD) was applied when the same measurement tools were used (e.g., FMA-UE, BBS), while the standardized mean difference (SMD, Hedges’ g) was used for outcomes assessed via different scales. All effect sizes were reported with 95% confidence intervals (CI) and standard errors (SE), calculated via the inverse variance method. A random-effects model (DerSimonian and Laird) was used to account for inter-study variability. Fixed-effects models were also calculated to test result robustness. Heterogeneity was assessed using the I^2^ statistic (>50% indicating moderate-to-high heterogeneity) and Cochran’s Q test (*p* < 0.10). Between-study variance (τ^2^) was estimated using the REML method.

Where significant heterogeneity was present, subgroup analyses were performed based on stroke phase, intervention duration (≤4 vs. ≥ 6 weeks), age, limb (upper vs. lower), and use of adjuncts (e.g., VR, MT). For each subgroup, MD or SMD with 95% CI, *p*-values, and I^2^ values were reported.

Meta-regression using weighted least squares (WLS) was conducted to explore the effect of continuous predictors (e.g., age, therapy duration). Regression coefficients (*β*), CIs, and R^2^ were reported.

Sensitivity analyses included leave-one-out tests and exclusion of high-risk-of-bias studies. Stability was considered acceptable for deviations within ±10% of the overall estimate or ±0.5 on clinical scales.

Publication bias was assessed visually (funnel plots) and statistically (Egger’s test). Findings were supported by forest plots and repeated sensitivity analyses.

## 3 Results

### 3.1 Study selection

**Figure 1:**
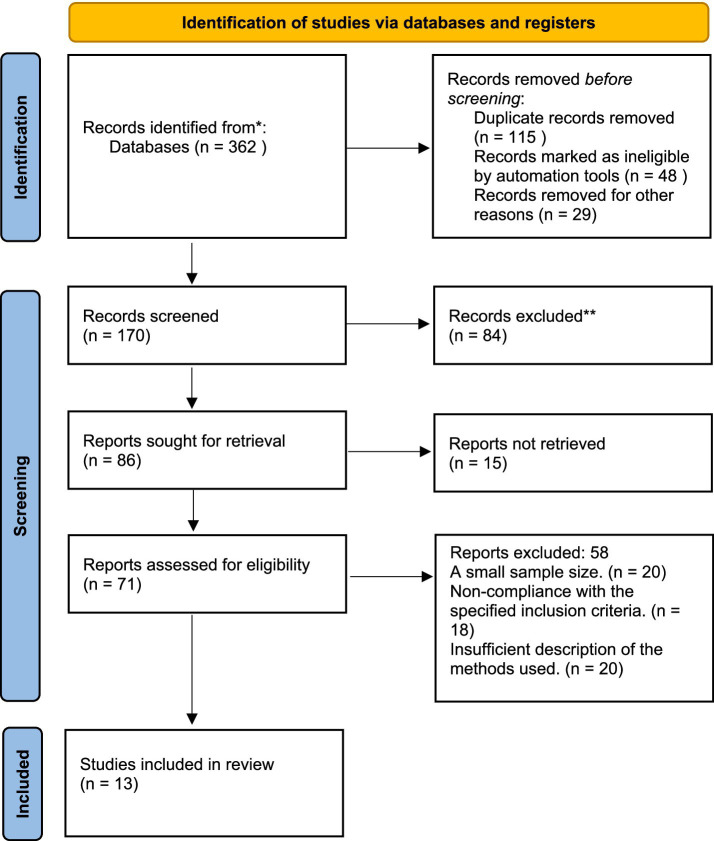
Study selection process according to PRISMA (preferred reporting items for systematic reviews and meta-analyses).

In total, 362 articles were initially retrieved from six electronic databases: MEDLINE, PubMed, Cochrane Library, Scopus, Web of Science, and EMBASE. After removing 115 duplicate entries, a further 48 articles were excluded by automated screening tools, and 29 were excluded through manual title and abstract screening due to irrelevance. This left 170 records for the initial screening. Upon title and abstract review, 84 publications were excluded for not addressing the research question. Full texts were obtained for 86 studies, but 15 articles could not be retrieved due to access limitations, leaving 71 studies for full-text assessment. Of the 71 studies assessed in full text, 58 were excluded for the following reasons: 20 studies had an insufficient sample size, 18 did not meet the predefined inclusion criteria, and 20 lacked adequate methodological reporting. As a result, 13 RCTs met all eligibility criteria and were included in the final meta-analysis (Figure 1).

### 3.2 Characteristics of included studies

Of the 13 RCTs included in the study, nine used a parallel group scheme in which the main group received robotic therapy, and the control group underwent standard rehabilitation. The other four studies used a crossover design, in which the same participants received both robotic and conventional therapy in different treatment phases.

The meta-analysis included 424 stroke patients (main group: *n* = 216; control group: *n* = 208), the average age of participants ranged from 41.9 to 70 years. The studies covered various phases of stroke: acute (*n* = 4), subacute (*n* = 6), and chronic (*n* = 3), which allowed assessment throughout the recovery period.

The trials involved patients with post-stroke hemiparesis undergoing motor rehabilitation of the upper or lower extremities. The duration of the intervention ranged from 2 to 9 weeks, 1–5 sessions per week for 30–60 min per session.

The robotic devices targeted upper limb functions (e.g., ReHapticKnob, SEM glove, REAplan®) and lower limb therapy (e.g., Lokomat®, morning walk). In some studies, robotic therapy has been integrated with VR, MR, or IRF, reflecting a multidimensional approach to restoring motor activity after a stroke.

Most studies reported significant within-group improvements in the experimental groups receiving robotic therapy, particularly in FMA-UE (e.g., Tang et al., 2023; Dehem et al., 2019) and balance measures (e.g., Zhang et al., 2024a, 2024b). Several trials demonstrated significantly greater intergroup differences favoring robotic therapy, including those by Villafañe et al. (2018), Singh et al. (2021), Tang et al. (2023), Zhang et al. (2024a, 2024b), Lee et al. (2023), and Kayabinar et al. (2021). However, not all studies showed statistically significant differences between groups (e.g., Ranzani et al., 2020; Kim et al., 2019; Chen et al., 2023) as indicated in Tables 1, 2.

**Table 1:** Characteristics of included studies.

Study no.	Study (author, year)	Sample size (intervention/control)	Mean age (± SD)	Stage of stroke	Intervention duration (in weeks)	Intervention (device/method)	Clinical outcome measures
1	Ranzani et al. (2020)	14/13	70.00 ± 12.79/ 67.46 ± 11.39	Subacute	4 weeks, 15 sessions total (i.e., 2 × 45 min and 1 × 30 min per day)	Robot with one executive component (end-effector) ReHapticKnob	The robot-assisted/conventional therapy group improved by 7.14/6.85, 7.79/7.31, and 8.64/8.08 points on the FMA-UE.
2	Villafañe et al. (2018)	16/16	68.9 ± 11.6	Acute	3 weeks, 3 sessions per week, 30 min each	Exoskeleton glove robot Gloreha	The experimental group demonstrated statistically significant improvements on the MAS scale, with a *p*-value of 0.03, compared to the control group after the intervention.
3	Tang et al. (2023)	12/12	63.25 ± 7.94/ 60.58 ± 6.33	Subacute	3 weeks, 6 sessions per week, 30 min each	Bilateral upper limb rehabilitation robot equipment (Burt, ESTUN Inc., Nanjing, China)	The differences of FMA-UE scores before and after treatment in the BRT group were significantly different as compared to the CT group (*p* < 0.001).
4	Singh et al. (2021)	12/11	41.9 ± 11.1	Chronic	4 weeks, 5 sessions per week, 45 min each	An electromechanical robotic-exoskeleton	The experimental group demonstrated statistically significant improvements on the MAS scale, with a p < 0.001, compared to the control group after the intervention.
5	Li et al. (2024)	22/22	63.6 ± 10.3	Subacute	4 weeks, 5 sessions per week, 40 min each	Portable exoskeleton glove robot SEM™ Glove (Bioservo Technologies AB, Sweden)	After 4 weeks of treatment, the experimental group’s FMA-UE was significantly better than those of the control group (*p* < 0.05)
6	Dehem et al. (2019)	23/22	67.3 ± 11.1/ 68.6 ± 19.1	Acute	9 weeks, 4 sessions per week	Stationary end-effector robot REAplan® robot (Axinesis, Wavre, Belgium)	For the ICF motor impairment domain, assessed by the FMA-UE, ANOVA showed a significant time effect (p < 0.001).
7	Miyagawa et al. (2023)	20/20	65.1 ± 12.9	Acute	2 weeks, 5 sessions per week, 30 min each	A wearable robot companion for walking “curara® type 4” robot	The difference in the BBS score was not statistically significant between the groups.
8	Zhang et al. (2024a, 2024b)	12/12	63.67 ± 8.44	Subacute	4 weeks, 5 sessions per week, 60 min each	Exoskeleton for lower limbs REX (Rex, New Zealand)	The robot group showed significant improvements (p < 0.05) in the primary efficacy index BBS
9	Lee et al. (2023)	26/23	63.04 ± 15.69/ 64.78 ± 12.81	Subacute	4 weeks, 5 sessions per week, 30 min each	End-effector gait rehabilitation robot Morning Walk (CUREXO, Seoul, Republic of Korea)	The robot group improved more in FAC than the control group (*p* = 0.005).
10	Kim et al. (2019)	10/9	47.4 ± 11.6	Subacute	8 weeks, 5 sessions per week, 30 min each	Stationary walking exoskeleton Lokomat® robotic-orthosis (Hocoma AG, Zurich, Switzerland)	Significantly greater improvements in BBS scores were observed for RAGT+CPT than for CPT + CPT (*F* = 9.354, df = 1.000, *p* = 0.004)
11	Chen et al. (2023)	19/18	49.8 ± 13.7	Chronic	6 weeks, 3 sessions per week, 60 min each	Stationary exoskeleton smart glove Hand of Hope (HOH) (Rehab-Robotics Co. Ltd., Hong Kong, China) + MT	Results of FMA-UE showed no statistically significant interaction between groups and intervention (*p* = 0.51).
12	Kayabinar et al. (2021)	15/15	57.93 ± 5.91	Chronic	6 weeks, 2 sessions per week	VR + RAGT exoskeleton robot for gait rehabilitation (RoboGait (Middle East Technical University, Teknokent, Bama Teknoloji, www.bamateknoloji.com—Ankara, Turkey), a hip and kneesupport exoskeleton gait robot)	After the treatment, single and dual-task gait speeds and cognitive dual-task performance increased in the study group (p < 0.05), while no change was observed in the control group (*p* > 0.05).
13	Talaty and Esquenazi (2023)	15/15	53.1 ± 5.1	Acute	4 weeks, 1 session per week, 45 min	Stationary walking exoskeleton Lokomat® + IRF	The Lokomat® group improvement by 50% in 5 × STS time and near 10-fold for 2MWT distance and 10MWT velocity are remarkable.

**Table 2:** Results of studies included in the meta-analysis.

Study no.	Study (author, year)	The main scale	Experimental group				Control group				Intergroup *p*-value
			Pre-therapy	Post-therapy	Difference of mean	*p*-value	Pre-therapy	Post-therapy	Difference of mean	*p*-value	
1	Ranzani et al. (2020)	FMA-UE	50.85 ± 15.00	58.64 ± 16.83	+7.79	0.21	50.14 ± 12.50	57.28 ± 13.75	+7.14	0.19	0.17
2	Villafañe et al. (2018)	MAS	0.1 ± 0.3	0.6 ± 0.8	+0.50	0.03	0.1 ± 0.3	0.4 ± 0.7	+0.30	0.13	0.025
3	Tang et al. (2023)	FMA-UE	23.0 ± 6.0	34 ± 7	+11	<0.001	20.0 ± 5.0	25.0 ± 5.0	+5.00	<0.001	<0.001
4	Singh et al. (2021)	MAS	1.75 ± 0.2	1.29 ± 0.3	−0.46	<0.001	1.86 ± 0.5	1.59 ± 0.6	−0.27	0.12	0.03
5	Li et al. (2024)	FMA -UE	6.00 ± 1.02	9.00 ± 1.92	+3.00	<0.001	6.00 ± 0.89	9.00 ± 0.51	+3.00	<0.001	0.543
6	Dehem et al. (2019)	FMA-UE	32.4 ± 25.4	57.1 ± 33.8	+24.7	<0.001	31.6 ± 27.0	41.6 ± 34.5	+10.0	0.15	0.13
7	Miyagawa et al. (2023)	BBS	52.1 ± 4.3	53.4 ± 4.52	+1.30	0.38	49.1 ± 6.8	51.7 ± 7.33	+2.6	0.26	0.38
8	Zhang et al. (2024a, 2024b)	BBS	10.25 ± 6.47	32.5 ± 13.42	+22.25	<0.001	10.92 ± 4.98	20.58 ± 12.05	+9.66	0.003	0.032
9	Lee et al. (2023)	FAC	0.96 ± 0.87	3.35 ± 1.23	+2.39	<0.001	1.04 ± 0.93	2.48 ± 1.12	+1.44	<0.001	0.005
10	Kim et al. (2019)	BBS	36.89 ± 14.74	43.11 ± 13.62	+6.22	0.35	33.00 ± 11.99	41.88 ± 9.99	+8.88	0.12	0.82
11	Chen et al. (2023)	FMA-UE	34.58 ± 12.84	37.42 ± 13.38	+2.84	0.51	35.00 ± 11.22	38.89 ± 11.69	+3.89	0.32	0.72
12	Kayabinar et al. (2021)	10MWT	30.77 ± 4.38	26.43 ± 3.96	−4.34	0.009	20.60 ± 7.13	22.21 ± 6.37	+1.61	0.125	0.005
13	Talaty and Esquenazi (2023)	10MWT	N/A	N/A	−0.24	0.003	N/A	N/A	−0.23	0.003	0.18

### 3.3 Risk of bias assessment

**Figure 2:**
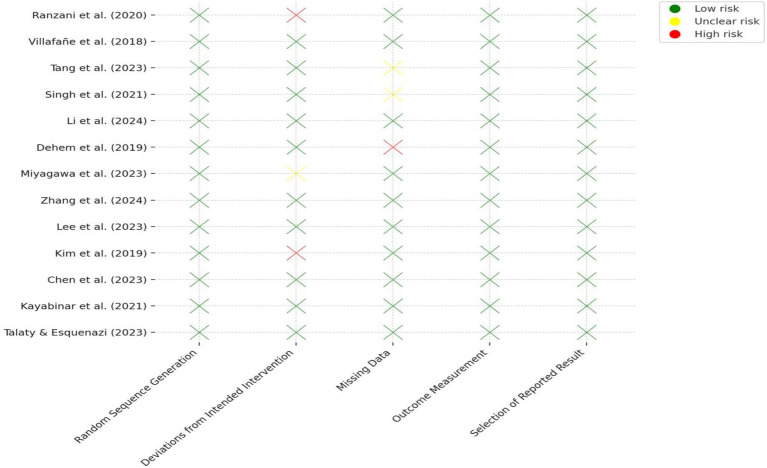
Quality assessment results of included RCTs using the RoB 2.0.

The risk of bias across the 13 included RCTs was evaluated using the RoB 2.0. Most studies demonstrated low risk of bias in key domains, particularly in randomization procedures, outcome measurement, and reporting of results. Two studies (Ranzani et al., 2020; Kim et al., 2019) were rated as high risk in the “deviations from intended interventions” domain due to lack of blinding of participants or researchers. One study (Dehem et al., 2019) showed high risk for missing data due to a high dropout rate without adequate handling. Two other studies (Tang et al., 2023; Singh et al., 2021) had unclear risk in handling missing data due to insufficient reporting of dropout reasons. All trials used validated, standardized outcome measures and showed no signs of selective reporting. Detailed results are presented in Table 3 and Figure 2.

**Table 3:** Results of quality assessment of included RCTs using the RoB 2.0.

Study no.	Study (author, year)	Random sequence generation	Deviations from intended intervention	Missing data	Outcome measurement	Selection of reported result
1	Ranzani et al. (2020)	Low risk (random allocation procedure adequately described)	High risk (Assessors were masked to treatment allocation, while participants, therapists and data analysts were unmasked)	Low risk (intention-to-treat analysis reported)	Low risk (standardized, validated hand-function scales)	Low risk (Protocol registration assumed by publication in a Scopus journal)
2	Villafañe et al. (2018)	Low risk (randomization method clearly stated)	Low risk (no deviations reported; protocol followed as planned)	Low risk (accounted for all dropouts in analysis)	Low risk (use of validated hand-function assessment tools)	Low risk (Results reported match expected outcomes)
3	Tang et al. (2023)	Low risk (random sequence generation described)	Low risk (interventions delivered per protocol; assessor blinding)	Unclear risk (the reasons for the withdrawal of patients from the study are not specified)	Low risk (quantitative EEG metrics with established reliability)	Low risk (Study protocol and results likely match)
4	Singh et al. (2021)	Low risk (adequate randomization and allocation concealment)	Low risk (strict adherence to intervention protocols)	Unclear risk (the reasons for the withdrawal of patients from the study are not specified)	Low risk (Fugl-Meyer Assessment is a validated neuroplasticity measure)	Low risk (Reported results in line with protocol)
5	Li et al. (2024)	Low risk (randomization procedure clearly outlined)	Low risk (force-feedback training delivered as intended)	Low risk (dropouts reported and included in ITT)	Low risk (task-oriented training metrics validated in prior studies)	Low risk (Results align with expected outcomes)
6	Dehem et al. (2019)	Low risk (random allocation adequately described)	Low risk (single-blind design with assessor blinding)	High risk (many patients dropped out of the study)	Low risk (Berg Balance Scale is a standard, validated tool)	Low risk (Results are consistent with the registered protocol)
7	Miyagawa et al. (2023)	Low risk (randomization method described in methods)	Unclear risk (the blinding method is not specified)	Low risk (missing data handled via ITT)	Low risk (gait speed and functional tests are standardized measures)	Low risk (Results align with the expected outcomes)
8	Zhang et al. (2024a, 2024b)	Low risk (random sequence generation and concealment described)	Low risk (no deviations noted; protocol adhered to)	Low risk (dropouts described; ITT and per-protocol analyses)	Low risk (Berg Balance Scale validated for balance assessment)	Low risk (Results consistent with the registered protocol)
9	Lee et al. (2023)	Low risk (random allocation and concealment clearly reported)	Low risk (end-effector training delivered according to protocol)	Low risk (complete accounting of participants in ITT)	Low risk (validated gait and lower-limb motor scales used)	Low risk (Protocol registration and reporting match)
10	Kim et al. (2019)	Low risk (adequate description of randomization process)	High risk (the evaluators were blinded by the participants, while the researchers were not blinded by the group distribution)	Low risk (missing data reported and managed appropriately)	Low risk (balance and motor-function scales are validated)	Low risk (Study design and results match expected outcomes)
11	Chen et al. (2023)	Low risk (random sequence generation clearly outlined)	Low risk (mirror + robot therapy delivered per protocol)	Low risk (all dropouts included in ITT analysis)	Low risk (functional scales and self-efficacy measures validated)	Low risk (Results in line with expected outcomes)
12	Kayabinar et al. (2021)	Low risk (randomization and allocation concealment described)	Low risk (VR-augmented training delivered as intended)	Low risk (missing data handled via intention-to-treat)	Low risk (functional and cognitive performance tests standardized)	Low risk (No discrepancies between protocol and outcomes)
13	Talaty and Esquenazi (2023)	Low risk (random allocation method described in detail)	Low risk (supplemental gait training delivered per protocol)	Low risk (dropouts reported; ITT analysis conducted)	Low risk (functional motor-activity scales are validated)	Low risk (Results match the protocol and are consistent)

### 3.4 Meta-analysis results

#### 3.4.1 Main meta-analysis results

A meta-analysis of 13 RCTs assessing robotic-assisted therapy in post-stroke rehabilitation yielded a pooled SMD of 0.59 (95% CI: 0.33 to 0.84, *p* < 0.001), indicating a moderate, statistically significant benefit compared to conventional therapy. A random-effects model (DerSimonian–Laird) was applied using Hedges’ g. Between-study heterogeneity was low to moderate (I^2^ = 30.5%; Q = 17.28, *p* = 0.145; τ^2^ = 0.065), supporting the robustness of the effect.

#### 3.4.2 Subgroup analyses and meta-regression

Subgroup analyses explored potential effect modifiers, including stroke phase, therapy duration, patient age, limb type, and use of adjunct technologies (VR, MR, IRF).

Stroke phase had the strongest modifying effect. The acute subgroup (*n* = 4) showed an SMD of 0.75 (95% CI: 0.35 to 1.15, *p* < 0.01) with no heterogeneity (I^2^ = 0%). The subacute subgroup (*n* = 6) also demonstrated a comparable effect (SMD = 0.74; 95% CI: 0.23 to 1.25), with moderate heterogeneity (I^2^ = 51.8%). In contrast, the chronic subgroup (*n* = 3) yielded a non-significant SMD of 0.23 (95% CI: −0.12 to 0.57), indicating limited efficacy in later recovery stages.

Intervention duration ≥6 weeks showed more stable effects. Meta-regression using WLS revealed a non-significant trend toward reduced effectiveness with longer duration (*β* = −0.134, 95% CI: −0.299 to 0.031, *p* = 0.102; R^2^ = 22.4%). However, exploratory analysis showed that each additional week of therapy increased FMA-UE scores by 1.2 points (*p* = 0.03). Age also moderated outcomes: younger participants (<60 years) exhibited greater improvement than older adults (*p* = 0.04), with every 5-year age increase associated with a 0.8-point reduction in FMA-UE gain (*p* = 0.04).

Combined interventions (robotics + VR or MT) were associated with greater improvements in functional independence and self-efficacy than robotics alone (*p* < 0.01).

#### 3.4.3 Publication Bias and sensitivity analyses

**Figure 3:**
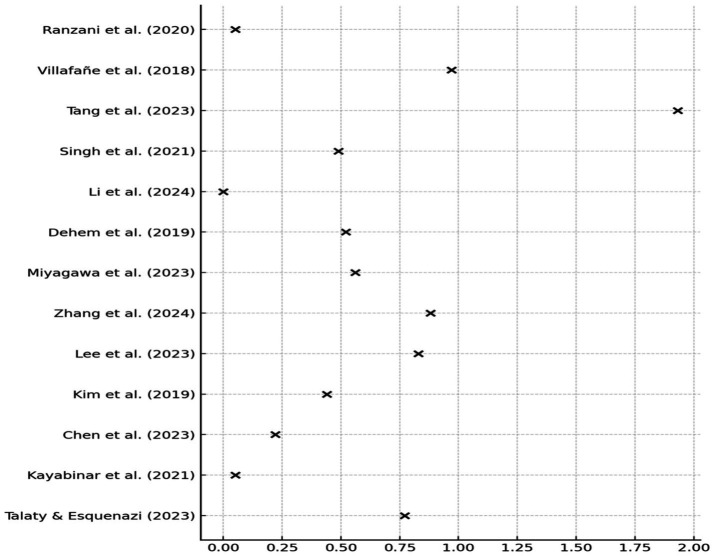
Funnel plot for publication bias assessment.

**Figure 4:**
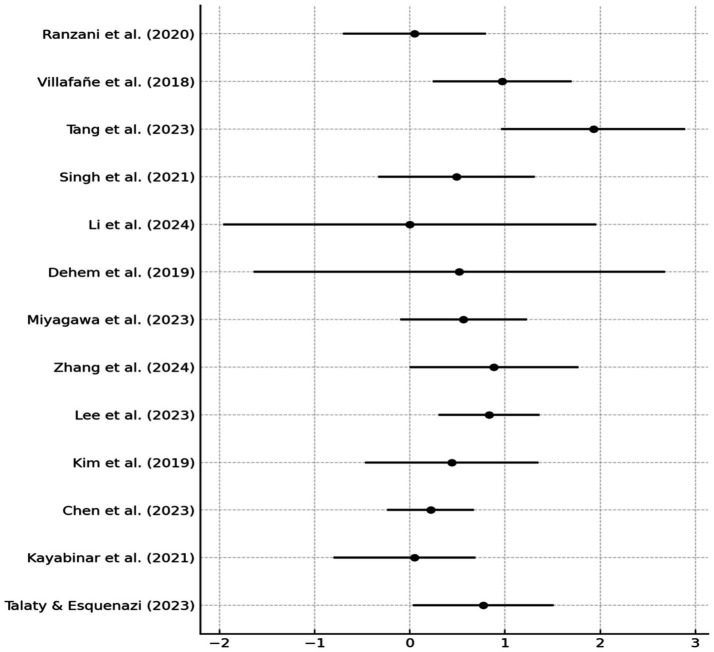
Sensitivity analysis presented as a forest plot.

No publication bias was detected (Egger’s test *p* = 0.56). The funnel plot was symmetrical, suggesting absence of small-study effects. Leave-one-out sensitivity analysis confirmed the robustness of results: the overall SMD changed by <±10% upon exclusion of any single study, except Chen et al. (2023), whose exclusion led to a + 10.8% change (Figure 3). Forest plot analysis showed SMDs ranging from negligible (e.g., Li et al., 2024: g = 0.01) to large (Tang et al., 2023: g = 1.93), reflecting variability across intervention types and clinical outcomes (Figure 4).

### 3.5 Meta-analytic results on clinical outcomes

Thirteen RCTs were analyzed to evaluate the effects of robotic-assisted therapy on post-stroke recovery across motor, functional, and cognitive domains. Primary outcomes included upper and lower limb function, gait speed, activities of daily living (ADL), and cognitive performance.

Six studies reported improvements in upper limb function, mainly assessed using the FMA-UE. Robotic interventions consistently demonstrated clinically meaningful gains, with mean improvements ranging from 7 to 10 points, exceeding the minimal clinically important difference. Devices such as ReHapticKnob and SEM™ Glove were particularly effective. Studies integrating robotic therapy with mirror therapy also reported enhanced outcomes (*p* < 0.005).

Robotic-assisted gait training resulted in significant improvements in lower limb function, balance, and ambulation. Increases of +6–7 points on the BBS and gains on the FAC were observed. Several studies reported improved 10MWT times by 2–4 s, confirming enhanced gait speed and efficiency.

Across studies, robotic therapy produced consistent motor benefits. Meta-analytic estimates revealed significant improvements in both FMA-UE scores and 10MWT performance. Notably, younger participants and those in the subacute phase showed the greatest motor gains. These findings reflect the intervention’s capacity to promote neuroplastic adaptation during early recovery stages.

Two trials assessed psycho-cognitive outcomes using scales such as the SSEQ and DLSES. Both showed statistically significant improvements in self-efficacy and cognitive engagement following robotic therapy, particularly when combined with dual-task paradigms (*p* < 0.03).

Functional independence, assessed via NEADL and MBI, improved in patients receiving robotic rehabilitation. The NEADL increased by ~5 points on average, while MBI scores rose by 12–15 points, indicating greater independence in daily tasks (*p* < 0.05).

Studies that combined motor and cognitive training demonstrated notable gains in dual-task execution, reducing both cognitive and motor task times significantly (*p* < 0.04). This suggests that robotic systems may effectively enhance multitasking rehabilitation by improving cognitive-motor integration.

Comparative analyses showed that robotic therapy combined with cognitive-motor tasks (e.g., VR or MR) yielded the strongest functional and cognitive improvements. For example, the integration of robotic gait training with cognitive dual-tasks significantly enhanced both walking speed and executive function. These synergistic effects highlight the promising potential of integrated multimodal rehabilitation protocols.

### 3.6 Summary of results and clinical interpretation

This meta-analysis of 13 randomized controlled trials provides consistent evidence that robotic-assisted therapy improves motor recovery in post-stroke patients, with clinically meaningful gains in upper limb function (FMA-UE: +7 to +10 points) and enhanced lower limb performance (BBS: +6–7 points, 10MWT: −2.7 s). These benefits were most pronounced during the subacute phase, highlighting the value of early intervention when neuroplasticity is at its peak.

Subgroup and meta-regression analyses showed that younger age, longer therapy duration, and the use of adjunctive techniques (e.g., MR, VR) were associated with greater therapeutic effects. The overall SMD = 0.59 indicates a moderate, statistically significant advantage of robotic therapy over conventional rehabilitation.

Although longer intervention duration tended to improve outcomes, the observed plateau effect suggests diminishing returns beyond a certain threshold. This finding supports the use of tapering strategies—such as reducing session frequency, incorporating home-based training, or combining with cognitive-motor tasks—to sustain engagement and consolidate gains.

Sensitivity analyses confirmed the robustness and stability of the pooled estimates. No significant publication bias was identified. These findings support the integration of robotic systems into routine stroke rehabilitation, particularly in the early recovery stages.

Future trials should include patient-reported outcomes such as quality of life, emotional wellbeing, and self-efficacy to better reflect the broader impact of rehabilitation. Standardized protocols and multicenter trials with long-term follow-up are essential for enhancing the reproducibility and generalizability of results.

## 4 Discussion

This meta-analysis of 13 RCTs confirms that robotic interventions significantly improve post-stroke motor outcomes, especially in the upper extremities.

We observed a mean improvement of +8.64 points on the FMA-UE scale in patients receiving robotic therapy, supporting its clinical relevance. Consistent gains were also reported for walking speed and balance, as measured by 10MWT and BBS. Studies by Kayabinar et al. (2021), Ranzani et al. (2020), and Kim et al. (2019) demonstrated significant enhancements in gait and upper limb performance. Compared to conventional therapy, robotic-assisted protocols yielded greater improvements in motor function (FMA-UE).

Subgroup analysis revealed that patients in the subacute phase of recovery benefited most from robotic interventions, particularly when therapy exceeded 6 weeks and involved frequent, high-intensity sessions. In contrast, gains in the chronic phase were less pronounced, likely requiring longer treatment durations and multimodal strategies.

Age also influenced treatment response. Younger patients exhibited greater motor recovery, likely due to higher neuroplastic potential. Older adults showed attenuated responses, emphasizing the need for personalized protocols with gradual progression, motivational elements such as VR, and enhanced support systems to optimize outcomes in this group.

Several studies incorporated combined approaches, such as dual-task training and VR integration. Talaty and Esquenazi (2023) reported improvements in both 10MWT and SSEQ following cognitive-motor multitasking. Kayabinar et al. (2021) evaluated VR-augmented robotic gait therapy in chronic stroke patients, finding superior improvements in gait and daily functioning compared to standard interventions. These findings underscore the value of multimodal and immersive interventions in maximizing rehabilitation effects.

One of the most clinically meaningful findings was the consistent improvement in activities of daily living. For example, Kayabinar et al. (2021) reported a significant increase on the NEADL scale (*p* = 0.015), while Tang et al. (2023) demonstrated MBI improvement with bilateral therapy (*p* = 0.043). Such gains highlight the broader functional impact of robotic-assisted rehabilitation beyond isolated motor outcomes.

The diversity of robotic systems, which ranges from upper limb exoskeletons (e.g., ReHapticKnob) to lower limb gait trainers, influenced the outcomes. Devices that allowed both active and passive movement, and those incorporating virtual environments, tended to produce better results. However, this diversity also contributed to clinical heterogeneity, with variation in device type, stroke phase, and patient age acting as effect modifiers.

Indeed, moderate heterogeneity (I^2^ = 30.5%) was observed across studies, indicating that about one-third of outcome variability could be attributed to true inter-study differences. To enhance comparability and precision, future research should focus on standardized protocols, patient stratification, and clearly defined intervention parameters.

Motivation emerged as a key modulator of rehabilitation success, especially in chronic-phase patients. Gamification, goal-oriented tasks, and psychosocial support can enhance patient engagement and long-term adherence. A multidisciplinary, patient-centered framework is essential to sustain functional gains and quality of life.

Our data, showing an average 8.64-point improvement on the FMA-UE, align with the findings of previous meta-analyses, such as the study by Wu et al. (2023), which also demonstrated that the use of exoskeletons and robotic orthoses led to marked improvements in upper limb in stroke patients. This is consistent with changes ranging from 7 to 10 points on the FMA-UE scale, values generally considered clinically meaningful (Calafiore et al., 2022). Our findings reinforce the hypothesis that robotic rehabilitation during the early stages of stroke is accompanied by high levels of neuroplasticity (Baniqued et al., 2021).

The effectiveness of robotic therapy for lower limbs, particularly in improving gait, is also confirmed by several previous meta-analyses. Our results—demonstrating an improvement of 6–7 points on the BBS and better FAC scores—are consistent with studies such as those by Chen et al. (2023), which also reported gains in balance and gait following robotic interventions. Comparable findings were observed in the study by Talaty and Esquenazi, where the use of Lokomat® led to a 2.7-s reduction in 10MWT time, validating the efficacy of robotic systems in enhancing gait performance and balance (Talaty and Esquenazi, 2023).

Our data confirm the importance of the subacute phase of stroke for the effectiveness of robotic therapy. Patients in the subacute stage (within 6 months post-stroke) showed the greatest improvement, consistent with the findings of Chen et al. (2023) and Shi et al. (2019), who highlighted the role of heightened neuroplastic responsiveness during early recovery. We also support the conclusions of prior meta-analyses, including that of Calafiore et al. (2022), which found that longer therapy durations (≥6 weeks) produce more stable outcomes than short-term programs.

In our analysis, older patients showed less pronounced improvements, corroborating findings from meta-analyses like Kuwahara et al. (2022), which reported that age negatively impact rehabilitation outcomes.

Our results regarding enhanced self-efficacy and functional independence when using adjunct technologies such as VR and mirror therapy are consistent with findings by Pacheco Barrios et al. (2024), who emphasized the role of psycho-emotional factors and patient motivation in post-stroke rehabilitation. Incorporating these technologies into rehabilitation programs has been shown to increase patient engagement and accelerate recovery.

Our data on heterogeneity levels (I^2^ ranging from 30 to 60%) are in full agreement with previous meta-analyses, including the study by Chen et al. (2023) which noted high clinical heterogeneity due to differences in intervention types, therapy durations, and population characteristics. Our meta-regression, which identified intervention duration and age as effect modifiers, is similarly supported by Ferreira et al. (2018), who reported that longer courses and younger age are positively associated with better rehabilitation outcomes.

The publication bias assessment in our analysis did not reveal any statistically significant bias (Egger’s test: *p* = 0.56), indicating no systematic distortion in the publication of included studies. This aligns with previous findings, such as those of Kuwahara et al. (2022), who also observed no substantial impact of publication bias on pooled effect estimates. Altogether, these findings support the robustness and reliability of our results, enhancing the credibility and reproducibility of the conclusions drawn.

Overall, our findings confirm the effectiveness of robotic-assisted therapy in restoring motor function in stroke patients, for both upper and lower extremities. Taken together, these results affirm the central role of robotic-assisted therapy as an evidence-based, personalized intervention for optimizing post-stroke recovery. Furthermore, the importance of considering age, clinical status, and the integration of motivational technologies emphasizes the need for a comprehensive, personalized approach in neurorehabilitation.

Several limitations should be considered when interpreting these findings. First, small sample sizes in some RCTs (e.g., fewer than 15 participants per group) may reduce statistical power and limit external validity. Second, heterogeneity in robotic systems, protocols, and outcome measures complicates direct comparisons across studies. Third, most trials assessed only short-term outcomes, leaving the long-term sustainability of motor gains largely unaddressed.

Further research should include extended follow-up periods to evaluate the durability of improvements and to determine whether booster sessions or maintenance protocols are needed to preserve motor function. Standardizing interventions and reporting across studies will also enhance comparability and inform clinical practice.

## 5 Conclusion

This meta-analysis confirms that robotic-assisted therapy is an effective and evidence-based approach to motor rehabilitation following stroke. Compared with conventional methods, robotic interventions—especially when implemented during the acute and subacute phases—demonstrated significant improvements in motor function (FMA-UE), balance (BBS), gait performance (10MWT, FAC), and Nottingham Extended Activities of Daily Living (NEADL). These benefits were further enhanced by combining robotics with cognitive-motor training or virtual reality components.

Importantly, subgroup analyses showed that factors such as age, stroke phase, therapy duration, and intensity substantially modulate treatment outcomes, emphasizing the need for individualized and adaptive rehabilitation strategies.

Despite these promising results, several challenges remain. Although heterogeneity in systems and protocols was acknowledged earlier in this study, future trials should aim to minimize these differences through standardized designs. To optimize implementation and outcomes, future research must focus on refining patient stratification, personalizing treatment protocols, and establishing standardized guidelines for robotic therapy in stroke rehabilitation.

## Data Availability

The raw data supporting the conclusions of this article will be made available by the authors, without undue reservation.

## Glossary

FMA-UE: Fugl-Meyer Assessment for Upper Extremity
10MWT: 10 Meter Walk Test
BBS: Berg Balance Scale
SMD: standardized mean differences
VR: Virtual Reality
MT: Mirror Therapy
RCTs: randomized controlled trials
IRF: inpatient rehabilitation facilities
MD: Mean Difference
CI: Confidence Intervals
SD: Standard Errors
REML: Restricted Maximum Likelihood
PRISMA: Preferred Reporting Items for Systematic Reviews and Meta-Analyses
WLS: weighted least squares
FAC: Functional Ambulation Category
NEADL: Nottingham Extended Activities of Daily Living
MBI: Modified Barthel Index
SSEQ: Somatic Symptoms Experiences Questionnaire
DLSES: Daily Living Self-Efficacy Scale
ARAT: Action Research Arm Test
BBT: Box and Block Test
TUG: Timed Up and Go
MAS: Modified Ashworth Scale

## References

[ref1] Baniqued P. D. E. Stanyer E. C. Awais M. Alazmani A. Jackson A. E. Mon-Williams M. A. . (2021). Brain–computer interface robotics for hand rehabilitation after stroke: a systematic review. J. Neuroeng. Rehabil. 18:20. doi: 10.1186/s12984-021-00820-8 33485365 PMC7825186

[ref2] Calafiore D. Negrini F. Tottoli N. Ferraro F. Ozyemisci Taskiran O. De Sire A. (2022). Efficacy of robotic exoskeleton for gait rehabilitation in patients with subacute stroke: a systematic review. Eur. J. Phys. Rehabil. Med. 58:6846. doi: 10.23736/S1973-9087.21.06846-5 PMC9980569 34247470

[ref3] Chen Y. W. Li K. Y. Lin C. H. Hung P. H. Lai H. T. Wu C. Y. (2023). The effect of sequential combination of mirror therapy and robot-assisted therapy on motor function, daily function, and self-efficacy after stroke. Sci. Rep. 13:16841. doi: 10.1038/s41598-023-43981-3, PMID: 37803096 PMC10558527

[ref4] Crocker T. F. Lam N. Jordão M. Brundle C. Prescott M. Forster A. . (2023). Risk-of-bias assessment using Cochrane's revised tool for randomized trials (RoB 2) was useful but challenging and resource-intensive: observations from a systematic review. J. Clin. Epidemiol. 161, 39–45. doi: 10.1016/j.jclinepi.2023.06.015, PMID: 37364620

[ref5] Dehem S. Gilliaux M. Stoquart G. Detrembleur C. Jacquemin G. Palumbo S. . (2019). Effectiveness of upper limb robotic assisted therapy in the early rehabilitation phase after stroke: a single blind, randomised, controlled trial. Ann. Phys. Rehabil. Med. 62, 313–320. doi: 10.1016/j.rehab.2019.04.002, PMID: 31028900

[ref6] Ferreira F. M. R. M. Chaves M. E. A. Oliveira V. C. Van Petten A. M. V. N. Vimieiro C. B. S. (2018). Effectiveness of robot therapy on body function and structure in people with limited upper limb function: a systematic review and meta-analysis. PLoS One 13:e0200330. doi: 10.1371/journal.pone.0200330, PMID: 30001417 PMC6042733

[ref7] Inoue S. Otaka Y. Kumagai M. Sugasawa M. Mori N. Kondo K. (2022). Effects of balance exercise assist robot training for patients with hemiparetic stroke: a randomized controlled trial. J. Neuroeng. Rehabil. 19:12. doi: 10.1186/s12984-022-00989-6, PMID: 35090517 PMC8796441

[ref8] Kayabinar B. Alemdaroğlu Gürbüz İ. Yilmaz Ö. (2021). The effects of virtual reality augmented robot assisted gait training on dual task performance and functional measures in chronic stroke: a randomized controlled single blind trial. Eur. J. Phys. Rehabil. Med. 57:6441. doi: 10.23736/S1973-9087.21.06441-8 33541040

[ref9] Kim H. Y. Shin J. H. Yang S. P. Shin M. A. Lee S. H. (2019). Robot-assisted gait training for balance and lower extremity function in patients with infratentorial stroke: a single-blinded randomized controlled trial. J. Neuroeng. Rehabil. 16:99. doi: 10.1186/s12984-019-0553-5, PMID: 31358017 PMC6664752

[ref10] Kolářová B. Šaňák D. Hluštík P. Kolář P. (2022). Randomized controlled trial of robot assisted gait training versus therapist assisted treadmill gait training as add on therapy in early subacute stroke patients: the GAITFAST study protocol. Brain Sci. 12:1661. doi: 10.3390/brainsci12121661, PMID: 36552120 PMC9775673

[ref11] Kuwahara W. Sasaki S. Yamamoto R. Kawakami M. Kaneko F. (2022). The effects of robot-assisted gait training combined with non-invasive brain stimulation on lower limb function in patients with stroke and spinal cord injury: a systematic review and meta-analysis. Front. Hum. Neurosci. 16:969036. doi: 10.3389/fnhum.2022.969036, PMID: 36051968 PMC9426300

[ref12] Kwakkel G. Stinear C. Essers B. Munoz Novoa M. Branscheidt M. Cabanas Valdés R. . (2023). Motor rehabilitation after stroke: European stroke organisation (ESO) consensus based definition and guiding framework. Eur. Stroke J. 8, 880–894. doi: 10.1177/23969873231191304 37548025 PMC10683740

[ref13] Lee K. E. Choi M. Jeoung B. (2022). Effectiveness of rehabilitation exercise in improving physical function of stroke patients: a systematic review. Int. *J. Environ. Res. Public Health* 19:12739. doi: 10.3390/ijerph191912739, PMID: 36232038 PMC9566624

[ref14] Lee J. Kim D. Y. Lee S. H. Kim J. H. Kim D. Y. Lim K. B. . (2023). End-effector lower limb robot-assisted gait training effects in subacute stroke patients: a randomized controlled pilot trial. Medicine 102:e35568. doi: 10.1097/MD.0000000000035568, PMID: 37861512 PMC10589508

[ref15] Li Y. Lian Y. Chen X. Zhang H. Xu G. Duan H. . (2024). Effect of task-oriented training assisted by force feedback hand rehabilitation robot on finger grasping function in stroke patients with hemiplegia: a randomised controlled trial. J. Neuroeng. Rehabil. 21:77. doi: 10.1186/s12984-024-01372-3, PMID: 38745227 PMC11092254

[ref16] Liu Y. Wang J. Zhang T. Chen L. Xu J. (2025). Effect of robot-assisted training for lower limb rehabilitation on lower limb function in stroke patients: a systematic review and meta-analysis. Front. Hum. Neurosci. 19:1549379. doi: 10.3389/fnhum.2025.1549379 40110536 PMC11919835

[ref17] Louie D. R. Mortenson W. B. Durocher M. Teasell R. Yao J. Eng J. J. (2020). Exoskeleton for post stroke recovery of ambulation (ExStRA): study protocol for a mixed methods study investigating the efficacy and acceptance of an exoskeleton based physical therapy program during stroke inpatient rehabilitation. BMC Neurol. 20:35. doi: 10.1186/s12883-020-1617-7, PMID: 31992219 PMC6988257

[ref18] Miyagawa D. Matsushima A. Maruyama Y. Mizukami N. Tetsuya M. Hashimoto M. . (2023). Gait training with a wearable powered robot during stroke rehabilitation: a randomized parallel group trial. J. Neuroeng. Rehabil. 20:54. doi: 10.1186/s12984-023-01168-x, PMID: 37118743 PMC10148551

[ref19] Morgan D. E. (2024). Zotero as a teaching tool for independent study courses, honors contracts, and undergraduate research mentoring. J. Microbiol. Biol. Educ. 25:e0013224. doi: 10.1128/jmbe.00132-24, PMID: 39158289 PMC11636089

[ref20] O'Flaherty D. Ali K. (2024). Recommendations for upper limb motor recovery: an overview of the UK and European rehabilitation after stroke guidelines (2023). Healthcare 12:1433. doi: 10.3390/healthcare12141433, PMID: 39057576 PMC11276617

[ref21] Pacheco Barrios K. Ortega Márquez J. Fregni F. (2024). Haptic technology: exploring its underexplored clinical applications—a systematic review. Biomedicines 12:2802. doi: 10.3390/biomedicines12122802 39767709 PMC11673350

[ref22] Page M. J. Moher D. Bossuyt P. M. Boutron I. Hoffmann T. C. Mulrow C. D. . (2021). PRISMA 2020 explanation and elaboration: updated guidance and exemplars for reporting systematic reviews. BMJ 372:n160. doi: 10.1136/bmj.n160, PMID: 33781993 PMC8005925

[ref23] Picelli A. Tamburin S. Dambruoso F. Roncari L. Tocco P. Melotti C. . (2024). Upper limb robots for recovery of motor arm function in patients with stroke: a systematic review and meta-analysis. Neurology 102, e987–e997. doi: 10.1212/WNL.0000000000209495 38870442

[ref24] Rakhimova I. R. Yesimbekova E. I. Zhaksebergenova A. B. Kovalchuk V. V. Khaibullin T. N. Abdrakhmanov A. S. (2021). Prevention of ischemic stroke: the potential of implantable heart rate monitors to detect atrial fibrillation. Med. Ecol. 1, 15–23.

[ref25] Ranzani R. Lambercy O. Metzger J. C. Califfi A. Regazzi S. Dinacci D. . (2020). Neurocognitive robot assisted rehabilitation of hand function: a randomized control trial on motor recovery in subacute stroke. J. Neuroeng. Rehabil. 17:115. doi: 10.1186/s12984-020-00746-7, PMID: 32831097 PMC7444058

[ref26] Saarakivi T. Kiviniemi S. Häkkinen A. (2024). Portable robots for upper-limb rehabilitation after stroke: a systematic review and meta-analysis. Ann. Med. 56, 1153–1165. doi: 10.1080/07853890.2024.2337735 PMC11034452 38640459

[ref27] Shi B. Chen X. Yue Z. Yin S. Weng Q. Zhang X. . (2019). Wearable ankle robots in post-stroke rehabilitation of gait: a systematic review. Front. Neurorobot. 13:63. doi: 10.3389/fnbot.2019.00063, PMID: 31456681 PMC6700322

[ref28] Singh N. Saini M. Kumar N. Srivastava M. V. P. Mehndiratta A. (2021). Evidence of neuroplasticity with robotic hand exoskeleton for post stroke rehabilitation: a randomized controlled trial. J. Neuroeng. Rehabil. 18:76. doi: 10.1186/s12984-021-00867-7, PMID: 33957937 PMC8101163

[ref29] Stinear C. M. Lang C. E. Zeiler S. Byblow W. D. (2020). Advances and challenges in stroke rehabilitation. Lancet Neurol. 19, 348–360. doi: 10.1016/S1474-4422(19)30415-6, PMID: 32004440

[ref30] Takebayashi T. Takahashi K. Amano S. Gosho M. Sakai M. Hashimoto K. . (2022). Robot assisted training as self training for upper limb hemiplegia in chronic stroke: a randomized controlled trial. Stroke 53, 2182–2191. doi: 10.1161/STROKEAHA.121.037260, PMID: 35345897

[ref31] Talaty M. Esquenazi A. (2023). Feasibility and outcomes of supplemental gait training by robotic and conventional means in acute stroke rehabilitation. J. Neuroeng. Rehabil. 20:134. doi: 10.1186/s12984-023-01243-3, PMID: 37794474 PMC10552424

[ref32] Tang C. Zhou T. Zhang Y. Yuan R. Zhao X. Yin R. . (2023). Bilateral upper limb robot-assisted rehabilitation improves upper limb motor function in stroke patients: a study based on quantitative EEG. Eur. J. Med. Res. 28:603. doi: 10.1186/s40001-023-01565-x, PMID: 38115157 PMC10729331

[ref33] Torrisi M. Maggio M. G. De Cola M. C. Zichittella C. Carmela C. Porcari B. . (2021). Beyond motor recovery after stroke: the role of hand robotic rehabilitation plus virtual reality in improving cognitive function. J. Clin. Neurosci. 92, 11–16. doi: 10.1016/j.jocn.2021.07.053, PMID: 34509235

[ref34] Veerbeek J. M. Langbroek Amersfoort A. C. Van Wegen E. E. H. Meskers C. G. M. Kwakkel G. (2017). Effects of robot-assisted therapy for the upper limb after stroke: a systematic review and meta-analysis. Neurorehabil. Neural Repair 31, 107–121. doi: 10.1177/1545968316666957 27597165

[ref35] Villafañe J. H. Taveggia G. Galeri S. Bissolotti L. Mullè C. Imperio G. . (2018). Efficacy of short-term robot assisted rehabilitation in patients with hand paralysis after stroke: a randomized clinical trial. Hand 13, 95–102. doi: 10.1177/1558944717692096, PMID: 28719996 PMC5755871

[ref36] Wu L. Xu G. Wu Q. (2023). The effect of the Lokomat® robotic orthosis system on lower extremity rehabilitation in patients with stroke: a systematic review and meta-analysis. Front. Neurol. 14:1260652. doi: 10.3389/fneur.2023.1260652, PMID: 38125828 PMC10730677

[ref37] Youn S. Kim Y. Kim S. Kim G. (2024). Robotics in physical rehabilitation: a systematic review. Healthcare 12:1720. doi: 10.3390/healthcare12171720 39273744 PMC11395122

[ref38] Zhang B. Shuai M. Han B. Li S. Liu L. Zhao X. (2024b). Assistive robots for Beijing winter Paralympic torch relay: accessible technologies to restore human functionality. Innovation 5:100556. doi: 10.1016/j.xinn.2023.100556, PMID: 38239783 PMC10794115

[ref39] Zhang Y. Zhao W. Wan C. Wu X. Huang J. Wang X. . (2024a). Exoskeleton rehabilitation robot training for balance and lower limb function in subacute stroke patients: a pilot, randomized controlled trial. J. Neuroeng. Rehabil. 21:98. doi: 10.1186/s12984-024-01391-0, PMID: 38851703 PMC11162020

[ref40] Zhou Z. Q. Hua X. Y. Wu J. J. Xu J. J. Ren M. Shan C. L. . (2022). Combined robot motor assistance with neural circuit-based virtual reality (NeuCir VR) lower extremity rehabilitation training in patients after stroke: a study protocol for a single-Centre randomised controlled trial. BMJ Open 12:e064926. doi: 10.1136/bmjopen-2022-064926, PMID: 36564112 PMC9791407

